# Functional versus Nonfunctional Rehabilitation in Chronic Ischemic Stroke: Evidences from a Randomized Functional MRI Study

**DOI:** 10.1155/2016/6353218

**Published:** 2015-12-28

**Authors:** Maristela C. X. Pelicioni, Morgana M. Novaes, Andre S. C. Peres, Altay A. Lino de Souza, Cesar Minelli, Soraia R. C. Fabio, Octavio M. Pontes-Neto, Antonio C. Santos, Draulio B. de Araujo

**Affiliations:** ^1^Radiology Division, Department of Internal Medicine, Ribeirao Preto School of Medicine, University of Sao Paulo, 14049-900 Ribeirao Preto, SP, Brazil; ^2^Brain Institute/Onofre Lopes University Hospital, Federal University of Rio Grande do Norte, 59153-155 Natal, RN, Brazil; ^3^Department of Psychobiology, Federal University of Sao Paulo (UNIFESP), Sao Paulo, SP, Brazil; ^4^Department of Neuroscience and Behavior, Ribeirao Preto School of Medicine, University of Sao Paulo, 14049-900 Ribeirao Preto, SP, Brazil

## Abstract

Motor rehabilitation of stroke survivors may include functional and/or nonfunctional strategy. The present study aimed to compare the effect of these two rehabilitation strategies by means of clinical scales and functional Magnetic Resonance Imaging (fMRI). Twelve hemiparetic chronic stroke patients were selected. Patients were randomly assigned a nonfunctional (NFS) or functional (FS) rehabilitation scheme. Clinical scales (Fugl-Meyer, ARA test, and modified Barthel) and fMRI were applied at four moments: before rehabilitation (P1) and immediately after (P2), 1 month after (P3), and three months after (P4) the end of rehabilitation. The NFS group improved significantly and exclusively their Fugl-Meyer scores at P2, P3, and P4, when compared to P1. On the other hand, the FS group increased significantly in Fugl-Meyer at P2, when compared to P1, and also in their ARA and Barthel scores. fMRI inspection at the individual level revealed that both rehabilitation schemes most often led to decreased activation sparseness, decreased activity of contralesional M1, increased asymmetry of M1 activity to the ipsilesional side, decreased perilesional activity, and decreased SMA activity. Increased M1 asymmetry with rehabilitation was also confirmed by Lateralization Indexes. Our clinical analysis revealed subtle differences between FS and NFS.

## 1. Introduction

Stroke is the leading cause of disability and the second cause of death in the world [1]. Very often it leads to long-lasting disabilities, including motor and sensory deficits on one side of the body, as a result of injury in the contralateral hemisphere [2]. During the acute phase (<6 months), some motor functions may be recovered, which is often attributed to the reduction of cerebral edema and early neuronal plasticity [3]. However, about 60% of stroke survivors will maintain permanent motor deficits, especially in the upper limbs, and only 30% to 66% will be able to maintain or regain functionality of their paretic upper limb [4].

Among all motor rehabilitation strategies, physical therapy is still the most frequently used. Rehabilitation strategies in physical therapy are often based on active, active-assisted, or passive exercises and bilateral repetitive movements and often require strength. The exercises can be movements of articulation in a specific direction and have no functional purpose, for example, isolated motion of shoulder flexion. On the other hand, there are exercises that aimed at stimulating functional motor tasks, for example, picking up an object. Some functional exercises reproduce everyday motor functions and moreover there are functional approaches, such as neurodevelopmental techniques (NT) [5], which emphasize inhibition of abnormal muscle patterns or tone in order to facilitate functional and voluntary movements [6, 7]. Two examples of NT approaches are the Bobath and proprioceptive neuromuscular facilitation (PNF). Bobath emphasizes normalizing muscle tone and facilitating automatic and volitional movement by handling key body parts [8]. PNF, on the other hand, focus on using the intact or less paretic muscle groups to produce irradiation effects on more severely impaired groups. Furthermore, PNF involves patterns of movements, and many of them follow diagonal or spiral patterns and are directed with intention [9].

Functional approaches are largely used in clinical practice, although there are still not enough evidences of their eventual higher efficacy when compared to nonfunctional exercises [10–14]. Furthermore, the majority of these studies are based on clinical scales [15, 16] and lack information on neural mechanisms following rehabilitation.

Increasingly, noninvasive functional neuroimaging, such as functional Magnetic Resonance Imaging (fMRI), is becoming an important tool to evaluate poststroke functional reorganization. Overall, fMRI studies of poststroke motor reorganization have consistently reported increased activity of the primary motor cortex (M1) of the unaffected hemisphere [17–19]. Furthermore, different rehabilitation strategies have been associated with the reengagement of M1 activity of the affected hemisphere. However, it is not specific to the rehabilitation strategy used [20–24].

The present study aimed at using fMRI and three clinical scales (Fugl-Meyer, ARA test, and the Barthel index) to perform a longitudinal evaluation and comparison between two rehabilitation strategies: one based on NT, named functional strategy (FS) which simulates daily life activities, and the other based on a conventional nonfunctional strategy (NFS). We hypothesize that FS group will present a broader clinical improvement, accompanied by consistent patterns of cortical reorganization, as increased fMRI signal of the affected hemisphere, particularly of M1.

## 2. Methods

This study was approved by the Ethics and Research Committee of the University of Sao Paulo and individual written informed consent was obtained from all subjects.

### 2.1. Patients

Twelve chronic ischemic stroke survivors (aged between 38 and 71 years) were enrolled in this study (Table 1). The time of insult varied from 1 to 10 years, affecting the middle cerebral artery territory. All of them were stable in terms of their neurological deficits, and all had disproportionate hemiparesis with brachiofacial predominance. Clinical and demographical data (gender, age, stroke time, paresis side, and clinical characteristics) are shown in Table 1. Nine healthy volunteers (aged between 18 and 30 years, 2 women) formed the control groups of the study.

All patients met the following inclusion criteria: middle cerebral artery stroke confirmed either by Computed Tomography (CT) and/or Magnetic Resonance Imaging (MRI); ability to understand and perform the fMRI motor task; National Institute of Health Stroke Scale (NIHSS) between 1 and 5 [25]; and modified Rankin scale score between 2 and 3 [25]. Exclusion criteria were hemiplegia, dementia, difficulty to understand or to collaborate during rehabilitation, and spasticity index according to the Ashworth Modified Scale between 4 and 5.

### 2.2. Rehabilitation Strategies

Selected patients were randomized with respect to the rehabilitation strategy: functional (FS) versus nonfunctional (NFS). Both approaches were applied five times a week, for 30 sessions, 90 minutes each. Nonfunctional exercises were initiated by the proximal articulations and finalized in the distal articulations. These exercises did not reproduce motor functions similar to everyday use. Patients performed the movements in sitting, lying, and standing positions. The sequence was performed bilaterally and with repetitions. The number of repetitions was established in the first session based on each patient capacity to perform the exercises for 90 minutes without fatigue. From this moment on, as long as the patient presented an improvement, the number of repetitions increased gradually. That is, the patient performed faster the same sequence of exercises. At the beginning of treatment, patients spent about 40 seconds to perform each activity and 6 minutes to perform the entire sequence. NFS consisted of active or, when appropriate, assisted-active or passive movements of all upper limbs articulations in all directions (flexion, extension, abduction, adduction, internal rotation, external rotation, and circumduction of the shoulder; flexion and extension of the elbow; pronation and supination of the forearm; flexion, extension, radial deviation, and ulnar deviation of the wrist; flexion, extension, abduction, and adduction of the fingers) (see Supplementary Figure 3 in Supplementary Material available online at http://dx.doi.org/10.1155/2016/6353218). FS was based on Bobath, PNF, and movements simulating daily life activities involving upper limbs. Based on Bobath, we selected exercises to normalize muscle tone, such as rolling. Furthermore, the exercises evolved from simpler postures to positions that require greater motor control. The exercises were initiated in lying position and ended in a stand position. Based on PNF, we selected to our study movements that are functional and that are performed on the diagonal, such as playing with a tennis racquet, diagonal movement with a stick, and diagonal arm movement to pick up a ball. Furthermore, we selected movements that reproduce everyday motor functions like brushing hair, opening a door, and writing. FS used (i) rolling, performing abduction of the shoulder, extension of the elbow, extension of the fingers, and supination of the forearm to both sides; (ii) lying prone with elbow support, flexion of the elbows, pronation of the forearm, and abduction of the fingers (in this position, the patient trained to reach an object performing the movement on the diagonal); (iii) changing from prone position to cat position and then from cat position to sitting position; (iv) in a sitting position, patients raising a stick on the diagonal using both hands on both sides; (v) in a sitting position, patient performing movement of pinch with the fingers holding small objects, writing or drawing, playing cards, buttoning, and unbuttoning; (vi) in a sitting position, combing the hair; (vii) in standing position, playing ball with a tennis racket; (viii) in standing position, opening and closing a lock and performing pronation and supination of forearm. The number of repetitions was established with the same criterion of nonfunctional exercises. At the beginning of rehabilitation, patients spent an average of about 2 minutes and 50 seconds to perform each activity and 21 minutes to perform the entire sequence (Supplementary Figure 2).

### 2.3. Clinical Assessment

Clinical outcome was assessed by Fugl-Meyer scale for upper limb, Action Research Arm (ARA) test, and the modified Barthel index [26, 27]. Fugl-Meyer scale evaluates sensitivity, reflex, movement with and without synergy, speed, and coordination, with a three-point ordinal scale: (0) cannot perform, (1) partially achieved, and (2) performed completely. The ARA test is specific to functional activities, such as compression, gripping, clamping, and reaching, evaluated on a four-point scale: (0) no movement; (3) movement performed normally. Maximum score is 57 [26]. The modified Barthel index assesses the dependence of the individual to perform everyday activities. It provides information about difficulties related to eating, clothing, sphincter control (bladder and bowel), locomotion, and ambulation. It is a 10-item scale, with partial scores ranging from 0 (total dependence) to 15 (total independence). Scores higher than 60 indicate functional independence and the maximum score—100 points—demonstrates full independence [27].

We used fMRI and all clinical scales to evaluate the patients at four instants: before treatment (P1), immediately after rehabilitation (P2), at one month (P3), and at three months (P4) after the end of rehabilitation.

To evaluate the effect of time (P1 to P4) and group (FS versus NFS) in all scales, we used a repeated measures Generalized Linear Mixed Model (GLMM) in a 2 (group) × 4 (time) design, with dependent variables standardized by ranks prior to statistical analysis (SPSS v 18). Tukey post hoc comparisons were also performed. Data were presented with respect to median and interquartile range, and significance was set at *p* < 0.05.

### 2.4. Functional MRI

MRI were acquired in a 1.5 T scanner (Siemens, Magneton Vision) with a TX/RX head coil. fMRI consisted of 66 contiguous echo-planar (EPI) volumes, each with 16 axial slices (slice thickness = 6 mm; TR = 4600 ms; TE = 60 ms; flip angle = 90°; matrix = 64 × 64; FOV = 220 mm; voxel dimension = 3.44 mm × 3.44 mm × 6.00 mm). High-resolution anatomical images were also acquired using a T1-weighed GRE sequence with the following parameters: TR = 9.7 ms; TE = 4 ms; flip angle = 12°; matrix = 256 × 256; FOV = 256 mm; slice thickness = 1 mm; voxel dimension = 1 mm × 1 mm × 1 mm.

Prior to the fMRI session, subjects trained for the motor task. It consisted of opening and closing one of their hands at self-pace. fMRI paradigm followed a block design, alternating six blocks of rest (27 seconds each), with five blocks of unilateral hand movement (27 seconds each). There were two runs in each session, one for each hand. Patients were monitored to ascertain correct task execution, to count the number of repetitions in each task period, and to inspect for synkinesis. The duration of each fMRI session was 25 minutes.

fMRI processing was conducted in Brain Voyager QX (version 2.6). Preprocessing steps involved correction of motion artifact, slice time correction, and temporal filtering (using a high-pass filter at 0.01 Hz). Statistical analysis used the General Linear Model (GLM) with fixed effects. Hand movement was modeled with a boxcar function convolved with a double-gamma hemodynamic response function. The motion realignment parameters were used as nuisance predictors. False discovery rate (FDR) was used for multiple comparison correction, and significance was set at *q*[FDR] < 0.05. fMRI evaluation was based on careful visual inspection, made by two experienced fMRI researchers (Draulio B. de Araujo and Antonio C. Santos) [28, 29]. They were blind to the treatment allocation group of each subject.

### 2.5. Lateralization Index

Lateralization Index (LI) was based on two spherical volumes of interest (VOI) (*d* = 3 cm), positioned and centered at ipsilateral and contralateral M1. LI was calculated according to

(1) $$\begin{array}{c} LI=\frac{N_{c}-N_{i}}{N_{c}+N_{i}}, \end{array}$$

where *N*_*c*_ is the number of significant voxels (*q*[FDR] < 0.05) in the contralateral M1 (to the moving hand) and *N*_*i*_ in the ipsilateral hemisphere. Positive LI indicates asymmetric activity to the contralateral hemisphere, while negative LI values indicate asymmetric activity to the ipsilateral side. LI ~ 0 indicates symmetrical M1 activity. These values were extracted only for the paretic hand. For the control group, ipsilateral and contralateral M1 were defined with respect to the dominant hand.

A General Linear Mixed Model (GLMM) was used to evaluate the between-subjects effect (controls × patients) and also within-subject effects among patients (P1 to P4). Significance level was set at *p* < 0.05.

Pearson's correlation analysis was performed between changes in LI and in all scales (Fugl-Meyer, Barthel, and ARAT).

### 2.6. Predetermined Primary and Secondary Outcome

The primary outcome measure of the study was the difference of clinical scales scores between at baseline (P1) and immediately after (P2) the rehabilitation program, between groups. Secondary outcome measures included differences of clinical scales scores between at baseline (P1), after 1 month (P3), and after 3 months (P4) of the rehabilitation program. In addition we also aimed to evaluate the patterns of fMRI maps in each group.

## 3. Results

### 3.1. Clinical Scales

Baseline characteristics of both groups were compatible regarding gender, time of stroke, NIHSS, mRS, and Ashworth. There were no significant differences of clinical scales between groups, before rehabilitation.

**Figure 1:**
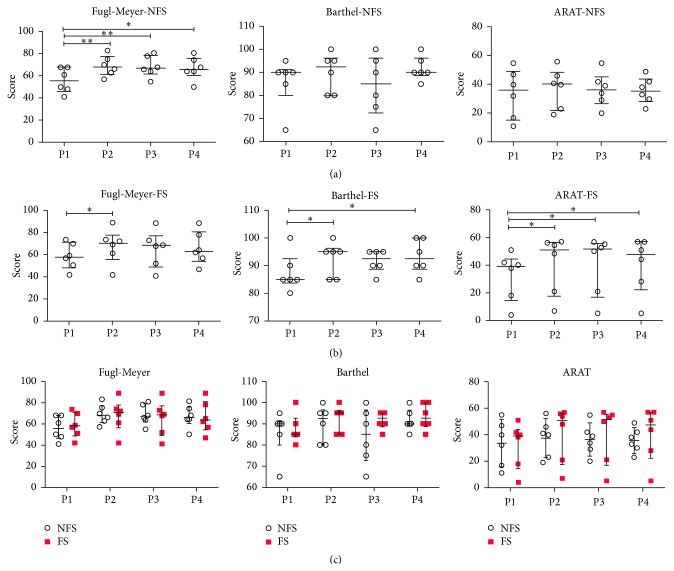
Results of all clinical scales (Fugl-Meyer, ARAT, and Barthel index), for the NFS and FS groups. Values are represented as median and interquartile interval. ^*∗*^ *p* < 0.05  ^*∗∗*^ *p* < 0.01 based on Tukey post hoc test. (a) shows the results for the clinical scales in the NFS group, before rehabilitation (P1), immediately after rehabilitation (P2), 1 month after the end of rehabilitation (P3), and three months after the end of rehabilitation (P4). (b) shows the results of clinical scales in the FS group. (c) shows the between-group comparison (NFS × FS) at the different periods of evaluation (P1, P2, P3, and P4).

An interaction effect group × time was found (Supplementary Table 1). For the NFS group, we observed differences only on the Fugl-Meyer scale, where scores at P1 were significantly smaller than at all other periods. For the FS group, differences on all three scales were found over time. In Figure 1, Tukey post hoc results show increment on ARAT scores from P1 to all other periods. Regarding Barthel scores, P4 and P2 were statistically different than P1, similar to what was found on Fugl-Meyer scale, which also has a significant increase from P2 with respect to P1.

Figure 1 also shows the between-group comparison (NFS × FS) at the different periods of evaluation. No significant difference was observed between groups (NFS × FS) at any period of evaluation.

### 3.2. Functional MRI

Six patients presented uncorrelated head movement or claustrophobia in at least one fMRI session, and data was analyzed only for the remaining periods. The other seven patients completed all fMRI evaluation successfully, in all sessions (P1, P2, P3, and P4).

At least one of five patterns was consistently observed as a result of rehabilitation, independently of the technique (FS × NFS): (i) decreased fMRI map sparseness, (ii) decreased activity of contralesional M1 (intact hemisphere), (iii) increased M1 activity in the ipsilesional side (damaged hemisphere), (iv) decreased perilesional activity, and (v) decreased SMA activity.

**Figure 2:**
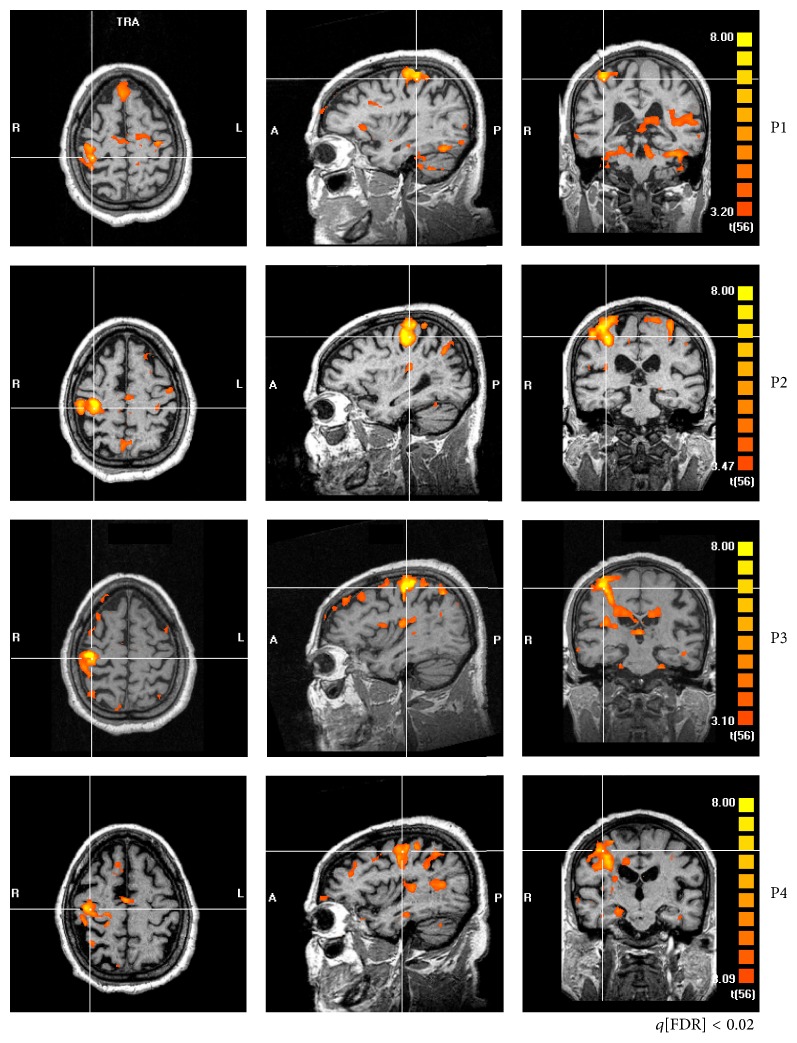
fMRI of a representative patient (#12) in all periods of evaluation (P1, P2, P3, and P4). Cross-lines are centered over M1 of the ipsilesional hemisphere. At P1, bilateral M1 activity, asymmetrical to the ipsilesional hemisphere. The maps are very sparse, particularly at P1. At P2, sparseness is reduced, and the activity is more confined to M1 and SMA. One month without rehabilitation (P3), the patterns become somehow similar to what they were before treatment onset, which is maintained three months after the end of treatment (P4).

An example is presented in Figure 2 (patient #12). At P1, the maps obtained from paretic hand movement are very sparse, including a number of nonmotor cortical structures, besides unusual bilateral activity of M1 and SMA. At the end of rehabilitation (P2), sparseness is reduced, and the activity is more confined to M1 and SMA of the contralateral hemisphere (ipsilesional). At one month (P3) as well as at 3 months without rehabilitation (P4), fMRI maps become similar to P1.

**Figure 3:**
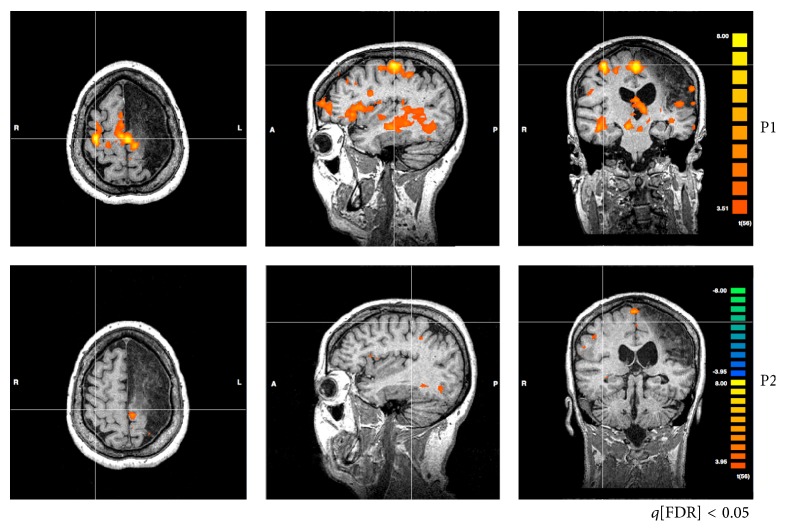
fMRI of patient #3 showing changes from contralesional M1 (at P1) to ipsilesional M1 (at P2). Before rehabilitation (P1), there is an increased activity of M1 in the contralesional hemisphere (ipsilateral to the moving hand) and of SMA. Right after rehabilitation, the activities of both areas are reduced.

Another consistent finding was the maladaptive increased activity of contralesional M1 (ipsilateral to the movement) found before rehabilitation (P1), which was related to motor performance. Patient #3, for instance, presented increased activity of contralesional M1, at P1, and subtle activity of ipsilesional M1. Increased ARAT score after training was related to a decrease of activity of both contralesional M1 and SMA (Figure 3).

Increased perilesional activity was also found prior to rehabilitation, for instance, patient #5 (Figure 5). After rehabilitation (P2), perilesional activity was reduced, together with higher Fugl-Meyer scores. This was not always the case, for instance, patient #1. fMRI at P1 shows no perilesional activity. However, after rehabilitation (at P2), increased perilesional activity was apparent, which was coincident with increased ARAT and Fugl-Meyer scores (Supplementary Figure 1).

Changes in SMA were also observed. For instance, decreased activity of this region was related to clinical improvement. Patients #1 and #12 improved their ARAT scores with a reduced activity of SMA (Figure 2 & Supplementary Figure 1).

**Figure 4:**
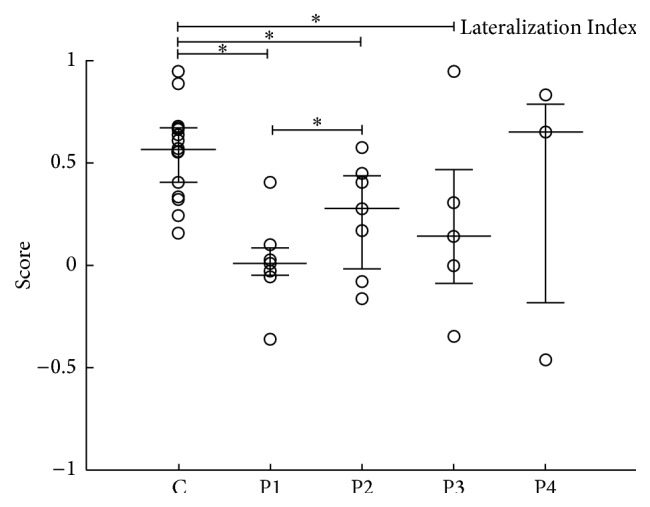
Lateralization Index (LI) of ipsilesional and contralesional M1. Values are represented as median and interquartile interval. LI of the control group is presented. For all patients, LI were evaluated at P1, P2, P3, and P4. ^*∗*^ *p* < 0.05; ^*∗∗*^ *p* < 0.01.

**Figure 5:**
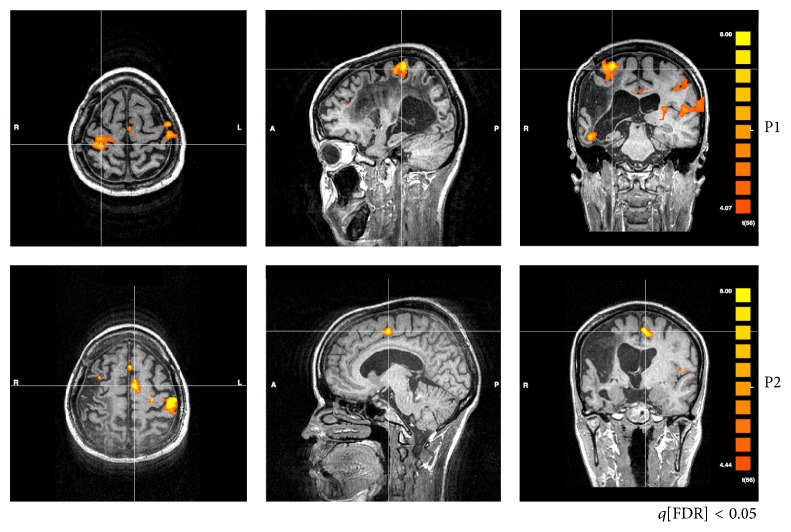
fMRI of patient #5 showing reduced perilesional activity at P2 with respect to P1. fMRI maps were obtained for the hand movements of the paretic hand. Images show decreased perilesional activity after rehabilitation.

Quantitative inspection of the contralateral and ipsilateral motor pathways was further based on the obtained LI values (Figure 4). We found an effect of time where patients were significantly different from controls in all periods (*p* = 0.001; P1, P2, and P3), except for P4. The Tukey post hoc test showed a significant difference only between P1 and P2, with respect to P1. LI values increased significantly at P2 (*p* = 0.03; becoming more asymmetric to the ipsilesional side) and decreased back again at P3 (*p* = 0.008).

No significant Pearson's correlation was found between LI and the three scales used: *r* = −0.6652 and *p* = 0.1031 (LI × Fugl-Meyer), *r* = 0.0971 and *p* = 0.8359 (LI × ARAT), and *r* = 0.0933 and *p* = 0.8422 (LI × Barthel) (Supplementary Figure 4).

## 4. Discussion

This study aimed at evaluating and comparing functional and nonfunctional rehabilitation strategies in ischemic stroke. Assessment was made with clinical scales and fMRI. Independent of the rehabilitation used, our results indicate that patients improve significantly at P2 (right after rehabilitation), in at least one clinical scale. Furthermore, the observed improvement persisted even without rehabilitation in the FS group, observed by both Barthel and ARAT, which evaluate fine movements. On the other hand, Fugl-Meyer scores decreased significantly after rehabilitation (both at P3 and at P4), particularly in the FS group (Figure 1).

As already pointed out in previous studies, a number of different rehabilitation techniques may improve motor functions of stroke survivors [30–32]. Our results reveal interesting specificities of each rehabilitation strategy. On the one hand, NFS are important when the main rehabilitation goal is to gain amplitude in a specific movement, rather than functionality. Therefore, its impact was observed exclusively by Fugl-Meyer scale. On the other hand, if therapy is focused on functional gain, particularly of fine movements, FS seems to be the choice. In fact, rehabilitation led to increased ARAT and Barthel scores, which persisted even after therapy.

The impact of rehabilitation on fMRI was marked by at least one of the following: decreased sparseness, particularly in the infarcted hemisphere, decreased activity of M1 of the intact hemisphere, and decreased SMA activity.

Consistent with our results (Figures 2 and 3), interhemispheric changes in the normal balance of M1 activity have been often observed in stroke, being more symmetrically distributed before rehabilitation [20, 33, 34]. Furthermore, our results also associated motor function improvement after rehabilitation with increased activity of contralateral M1 (Figure 3) [35, 36]. Such tendency was confirmed by our LI results, which indicates that symmetric activation of M1 (LI ~ 0) was predominantly found before rehabilitation, with increased asymmetry to the contralateral hemisphere of the moving hand after therapy (LI > 0).

There are increasing evidences that the observed activity of contralesional M1 is related to worse stroke recovery [18, 37–39]. These observations have also been present in rehabilitation schemes based on Constrained Induced Motion Therapy (CIMT) [40]. Clinical scales improvement was associated with decreased activity of contralesional M1 and increased ipsilesional M1 activity. Likewise, stroke survivors improved significantly Fugl-Meyer scores after mirror therapy, concomitant with increased ipsilesional M1 activity [21]. Herein, individual analysis indicates that higher ARAT scores are associated with increased ipsilesional M1 activity (e.g., patient #3).

Reduced sparseness also appears as a marker of clinical improvement in stroke survivors. For instance, longitudinal studies have shown that long-term training of specific tasks reduces the area of activity as detected by fMRI, for instance, following motor training [41, 42].

Additionally, our study found reduced activity of SMA, which was coincident with clinical improvement. Some studies support the idea that SMA activity is important for recovery [43, 44], and it has been suggested that increased activity of superior motor areas is related with reduced use of the affected arm [44].

Although our study found consistent clinical and fMRI changes related to rehabilitation, it is important to point out some of its limitations and caveats. First of all, the limited number of patients (6 in each group) hampers broader conclusion to the general population of stroke survivors. Furthermore, fMRI methods are based on unaltered cerebrovascular coupling, which is not the case in stroke [45–47]. Moreover, the lack of a control group (without any intervention) may limit our ability to attribute the observed improvement to the interventions, instead of natural history. Nevertheless, in our patients, the deficits were already at a plateau of functional capacity.

The search for new physical therapy techniques for patients with neurological deficits has been constant. The clinical outcome after rehabilitation can be measured by specific clinical scales and evaluates specific variables after treatment. In fact, our analysis revealed subtle differences between FS and NFS, indicating that the strategy of choice depends ultimately on the main goal to be achieved with rehabilitation. Furthermore, our fMRI results indicate some specific patterns that are best associated with the observed clinical improvements, which can be the focus of further investigation.

## Supplementary Material

Additional supporting information was added to the online version of article. Supplementary table 1 contains detailed information related to the analysis of clinical scales (Fugl Meyer, ARA-t and Barthel). Figure S1 shows the fMRI results of a patient with perilesional increased activity, together with improved ARA-t and Fugl-Meyer scores at P2, with respect to P1. Figures S2 and S3 were added to clarify the movements used during the Functional Strategy (suppl. Fig. 2), and Non-Functional Strategy (suppl. Fig. 3). Figure S4 brings the results of the correlation analysis between LI and the clinical scales (suppl. Fig. 4).

## Figures and Tables

**Table 1:** Demographic data, training type (FS × NFS), and clinical characteristics (paresis, NIHSS, Rankin, and modified Ashworth scales) of all patients at P1 (baseline, before intervention).

Patient	Sex	Age (y)	Intervention	Time of stroke (y)	Paresis	NIHSS	mRS	Ashworth
1	M	71	NFS	10	L	3	3	3
2	M	68	NFS	1,5	R	5	2	2
3	F	57	NFS	1	R	4	2	1
4	M	67	NFS	1,5	L	1	3	1
5	F	38	NFS	10	L	2	2	1
6	M	58	NFS	1	R	5	2	3
7	M	61	FS	9	R	3	2	3
8	M	48	FS	2	R	5	3	3
9	F	64	FS	1,5	R	5	2	3
10	M	69	FS	4,5	L	1	2	3
11	M	64	FS	1	R	3	3	3
12	M	59	FS	4,5	L	4	2	1

y: year, M: male, F: female, NFS: nonfunctional strategy, FS: functional strategy, L: left, R: right, NIHSS: National Institute of Health Stroke scale, mRS: modified Rankin scale.
