# Supplementary material for: Functional versus Nonfunctional Rehabilitation in Chronic Ischemic Stroke: Evidences from a Randomized Functional MRI Study
Source: Neural Plast. 2015 Dec 28;2016:6353218. doi: 10.1155/2016/6353218 (PMC4709724; doi:10.1155/2016/6353218)

**Supplementary table 1**. GLMM results of all clinical scales (Fugl Meyer, ARAt & Barthel), evaluated at all periods (P1, P2, P3 & P4). Confidence intervals were calculated upon the use of bootstrap with 1000 repetitions.

| group | time | scale | Mean | SD | 95% Confidence Interval | |
| --- | --- | --- | --- | --- | --- | --- |
|  |  |  |  |  | Lower Bound | Upper Bound |
| FS | p1 | Barthel | 87.5 | 3.7 | 79.3 | 95.7 |
|  |  | ARAt | 32.2 | 7.1 | 16.4 | 47.9 |
|  |  | Fugl-Meyer | 58.8 | 4.7 | 48.3 | 69.4 |
|  | p2 | Barthel | 92.5 | 3.0 | 85.8 | 99.2 |
|  |  | ARAt | 40.5 | 7.3 | 24.1 | 56.9 |
|  |  | Fugl-Meyer | 67.8 | 5.2 | 56.2 | 79.5 |
|  | p3 | Barthel | 91.7 | 4.0 | 82.8 | 100.6 |
|  |  | ARAt | 40.2 | 7.2 | 24.2 | 56.1 |
|  |  | Fugl-Meyer | 65.3 | 5.6 | 52.9 | 77.8 |
|  | p4 | Barthel | 93.3 | 2.3 | 88.2 | 98.5 |
|  |  | ARAt | 40.3 | 6.5 | 25.9 | 54.7 |
|  |  | Fugl-Meyer | 66.3 | 5.3 | 54.5 | 78.1 |
| NFS | p1 | Barthel | 85.8 | 3.7 | 77.7 | 94.0 |
|  |  | ARAt | 33.7 | 7.1 | 17.9 | 49.4 |
|  |  | Fugl-Meyer | 56.0 | 4.7 | 45.5 | 66.5 |
|  | p2 | Barthel | 90.0 | 3.0 | 83.3 | 96.7 |
|  |  | ARAt | 37.5 | 7.3 | 21.1 | 53.9 |
|  |  | Fugl-Meyer | 69.0 | 5.2 | 57.3 | 80.7 |
|  | p3 | Barthel | 84.2 | 4.0 | 75.3 | 93.1 |
|  |  | ARAt | 36.5 | 7.2 | 20.5 | 52.5 |
|  |  | Fugl-Meyer | 68.7 | 5.6 | 56.2 | 81.1 |
|  | p4 | Barthel | 91.7 | 2.3 | 86.5 | 96.8 |
|  |  | ARAt | 35.8 | 6.5 | 21.4 | 50.2 |
|  |  | Fugl-Meyer | 66.8 | 5.3 | 55.0 | 78.6 |

Figure S1. fMRI of patient #1 before (P1) and immediately after rehabilitation (P2). Perilesional activity increased at P2 (at the cross). The ARAt and Fugl-Meyer scores also improved at P2, with respect to P1.


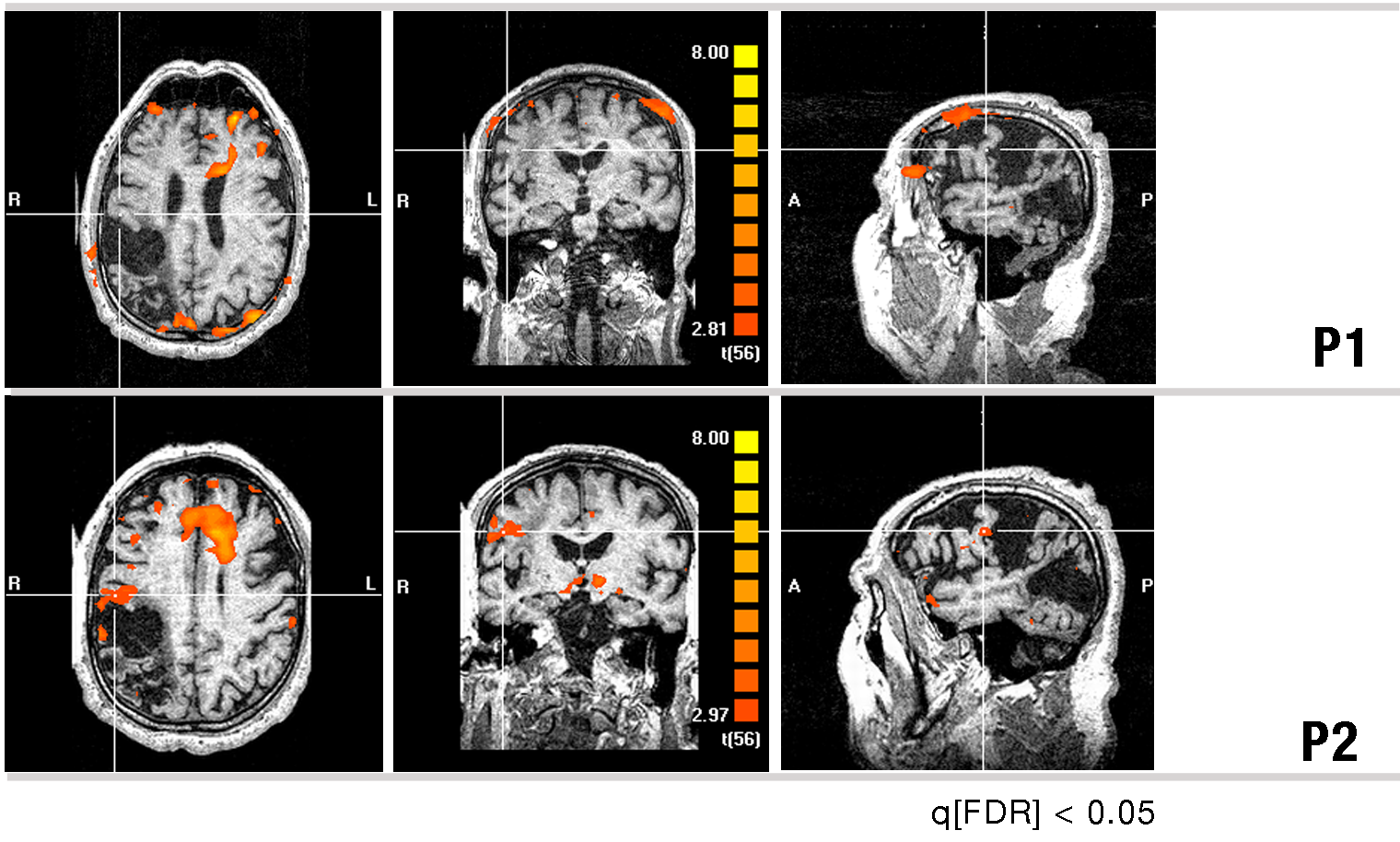


Figure S2. Movements used during the Functional Strategy


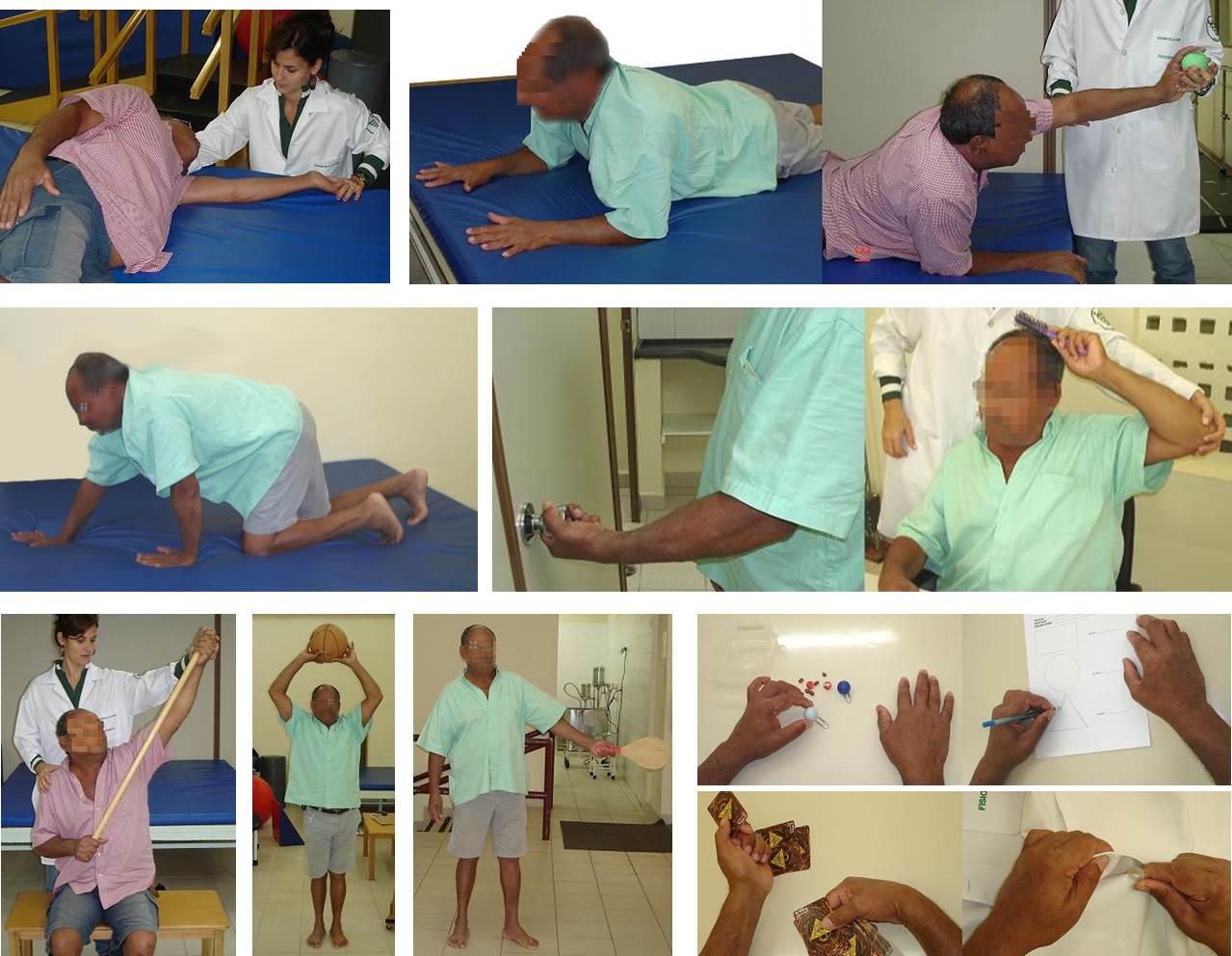


Figure S3. Movements used during the Non-Functional Strategy.

**
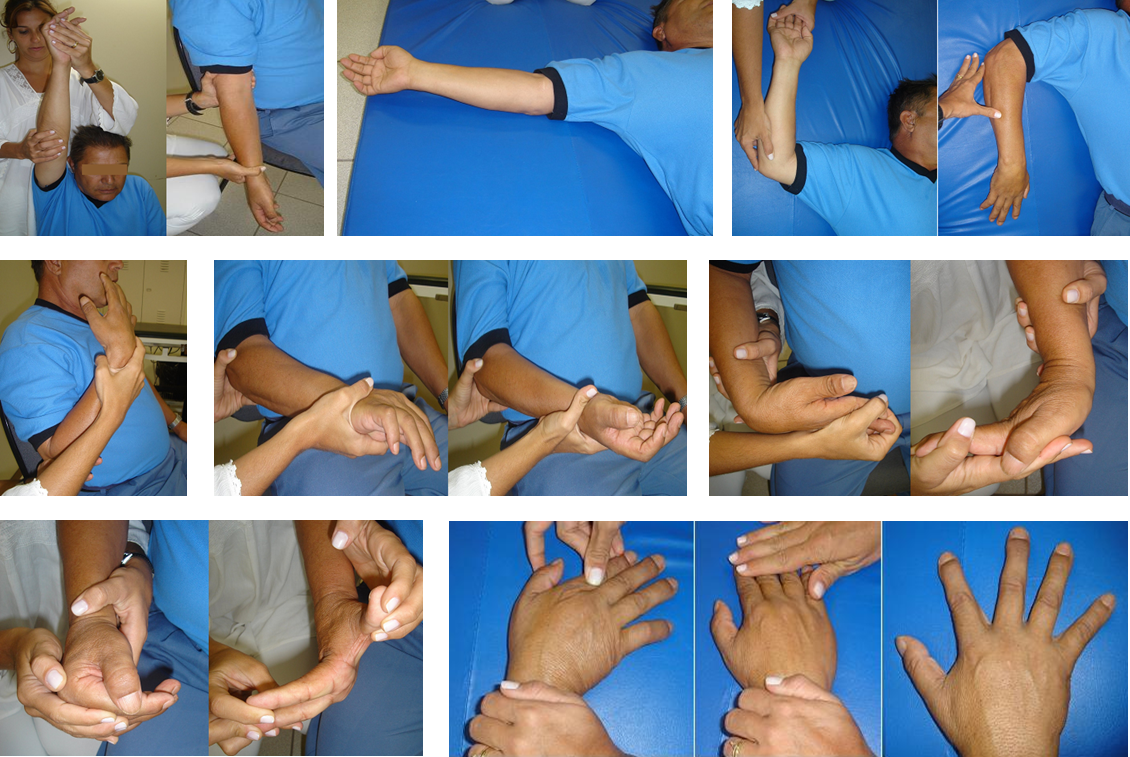
**

Figure S4. Pearson's correlation between Lateralization Index (LI) and scales (Fugl-Meyer, ARAT and Barthel). Values are represented as difference of LI (after - before) and scores in each scale (after - before). First row (a) correlation between LI and Fugl-Meyer; (b) correlation between LI and ARAT; (c) correlation between LI and Barthel. No significant difference was found.


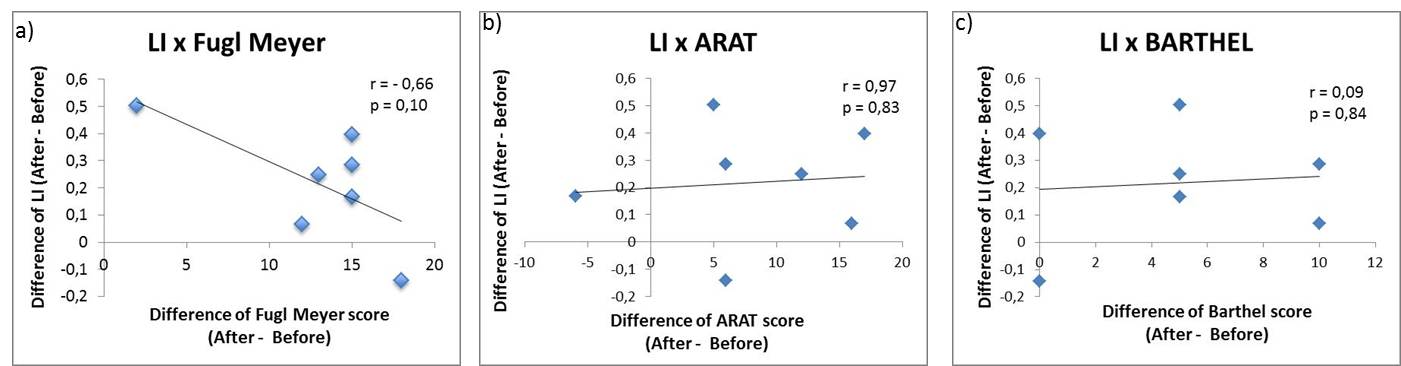

Supplement: Supplementary file 1 — Additional supporting information was added to the online version of article. Supplementary table 1 contains detailed information related to the analysis of clinical scales (Fugl Meyer, ARA-t and Barthel). Figure S1 shows the fMRI results of a patient with perilesional increased activity, together with improved ARA-t and Fugl-Meyer scores at P2, with respect to P1. Figures S2 and S3 were added to clarify the movements used during the Functional Strategy (suppl. Fig. 2), and Non-Functional Strategy (suppl. Fig. 3). Figure S4 brings the results of the correlation analysis between LI and the clinical scales (suppl. Fig. 4). [file 6353218.f1.docx]
